# Irreversible dual inhibitory mode: the novel Btk inhibitor PLS-123 demonstrates promising anti-tumor activity in human B-cell lymphoma

**DOI:** 10.18632/oncotarget.3824

**Published:** 2015-04-14

**Authors:** Ning Ding, Xitao Li, Yunfei Shi, Lingyan Ping, Lina Wu, Kai Fu, Lixia Feng, Xiaohui Zheng, Yuqin Song, Zhengying Pan, Jun Zhu

**Affiliations:** ^1^ Key Laboratory of Carcinogenesis and Translational Research (Ministry of Education), Department of Lymphoma, Peking University Cancer Hospital and Institute, Beijing, China; ^2^ Key Laboratory of Chemical Genomics, School of Chemical Biology and Biotechnology, Peking University Shenzhen Graduate School, Xili, Shenzhen, China; ^3^ Key Laboratory of Carcinogenesis and Translational Research (Ministry of Education), Department of Pathology, Peking University Cancer Hospital and Institute, Beijing, China; ^4^ Key Laboratory of Carcinogenesis and Translational Research (Ministry of Education), Central Laboratory, Peking University Cancer Hospital and Institute, Beijing, China; ^5^ Department of Pathology and Microbiology, University of Nebraska Medical Center, Omaha, NE, USA

**Keywords:** BCR signaling, Btk, irreversible inhibitor, targeted therapy, B-cell lymphoma

## Abstract

The B-cell receptor (BCR) signaling pathway has gained significant attention as a therapeutic target in B-cell malignancies. Recently, several drugs that target the BCR signaling pathway, especially the Btk inhibitor ibrutinib, have demonstrated notable therapeutic effects in relapsed/refractory patients, which indicates that pharmacological inhibition of BCR pathway holds promise in B-cell lymphoma treatment. Here we present a novel covalent irreversible Btk inhibitor PLS-123 with more potent anti-proliferative activity compared with ibrutinib in multiple cellular and *in vivo* models through effective apoptosis induction and dual-action inhibitory mode of Btk activation. The phosphorylation of BCR downstream activating AKT/mTOR and MAPK signal pathways was also more significantly reduced after treatment with PLS-123 than ibrutinib. Gene expression profile analysis further suggested that the different selectivity profile of PLS-123 led to significant downregulation of oncogenic gene PTPN11 expression, which might also offer new opportunities beyond what ibrutinib has achieved. In addition, PLS-123 dose-dependently attenuated BCR- and chemokine-mediated lymphoma cell adhesion and migration. Taken together, Btk inhibitor PLS-123 suggested a new direction to pharmacologically modulate Btk function and develop novel therapeutic drug for B-cell lymphoma treatment.

## INTRODUCTION

B-cell lymphoma is characterized by the malignant growth of B-lymphocytes in the lymph system and comprises various subtypes with distinct pathological characteristics, clinical features and prognoses [1]. In China, approximately 5-6 new lymphatic malignancy cases are diagnosed per 100,000 people per year, and B-cell lymphoma accounts for approximately 66% of these patients. Patient prognosis has been significantly improved by combination immunotherapy and rituximab; however, approximately 1/3 of late-phase patients still develop primary and secondary resistance to the chemotherapy [2]. Therefore, the development of new targeted drugs for the treatment of this debilitating disease is urgently needed.

The BCR signaling pathway has gained significant attention owing to its essential role in the pathogenesis of B-cell lymphoid malignancies [3]. BCR signaling transduction is initiated upon stimulation of the BCR complex, which is composed of surface immunoglobulin and two associated proteins: CD79A (Igα) and CD79B (Igβ). The CD79A and CD79B ITAM domains are phosphorylated by the Src-family kinases upon membrane-bound antigen stimulation, which triggers the phosphorylation and activation of regulatory and adaptor proteins, including Syk, Btk and B-cell linker (BLNK). BCR-mediated phosphorylation of the Tyr551 residue in Btk results in a 10-fold increase in Btk's catalytic activity. Then, this form of Btk undergoes auto-phosphorylation at Tyr223 to become fully activated. Once fully activated, Btk phosphorylates its substrates phospholipase C-γ2 (PLC-γ2) leading to the induction of downstream pathways, such as NF-κB, extracellular signal-regulated kinase (ERK) and NF-AT, which regulate B cell proliferation, differentiation, and migration [4]. Recently, several drugs targeting BCR signal, especially the Btk inhibitor ibrutinib (PCI-32765), have clearly demonstrated promising therapeutic effects in relapsed/refractory chronic lymphocytic leukemia (CLL) and B-cell non-Hodgkin lymphoma (B-NHL) [5-7]. Ibrutinib is the first selective and irreversible small-molecule Btk inhibitor assessed in clinical trials for the treatment of B-cell lymphoma. Ibrutinib interacts with the cysteine residue at position 481 in Btk (Cys481), which is located at the rim of the ATP-binding pocket. In a phase I, open-label trial, ibrutinib demonstrated notable anti-lymphoma activities and a good safety profile in B-cell malignancy patients. The overall response rate (ORR) of evaluable patients was 60%, including a complete response (CR) rate of 16%, which indicates that pharmacological inhibition of BCR pathway holds promise in B-cell lymphoma treatment [8, 9].

Investigating the biological function of Btk, de Rooij et al. reported that Btk activation is also regulated via a negative regulation loop that inhibition of Btk catalytic activity increases Tyr551 phosphorylation level [10, 11]. Thus, one direction for next-generation Btk inhibitors pursued by us was to inhibit both the catalytic activity and the activation of Btk by upstream kinases. By targeting both properties, these new inhibitors might potentially downregulate BCR and other related signal transduction pathways more effectively. Meanwhile, the different selectivity profile of these inhibitors might also offer new opportunities beyond what ibrutinib has achieved. Based on this hypothesis, we recently developed a novel series of covalent irreversible Btk inhibitors based on a Type II scaffold [12]. Two noticeable features of this series of inhibitors have emerged: (1) in contrast to ibrutinib, which only inhibits Btk's catalytic activity, these new inhibitors exhibit a dual-action mode of inhibition for Btk catalytic activity and its own activation; (2) this series of compounds presents a different selectivity profile from ibrutinib, which demonstrated potent activity against ABL, Blk, Bmx, Btk, EGFR, HER2, HER4, Lck, p38α, Rlk, Tec, and VEGFR [13, 14]. Among these compounds, PLS-123 displayed impressive potency against Btk and gave IC_50_ values of 13 nM against Btk Tyr551 and PLC-γ2 Tyr1217 phosphorylation.

In this paper, we demonstrated that this novel Btk inhibitor PLS-123 exhibited more potent anti-proliferative effects than ibrutinib in multiple cellular and *in vivo* preclinical models of B-cell lineage malignancy, including 14 kinds of cell lines, patients' primary tumor cells and mouse xenograft model. More importantly, the PLS-123′s potent efficacy against B-cell lymphoma might be attributed to effective apoptosis induction, dual-action Btk inhibitory mode and alternative selectivity profile offered additional anti-tumor mechanism. Collectively, a new direction was suggested to pharmacologically modulate Btk function and efficiently treat B-cell lymphoma.

## RESULTS

### PLS-123 inhibited the viability of B-cell lymphoma cells effectively

**Figure 1:**
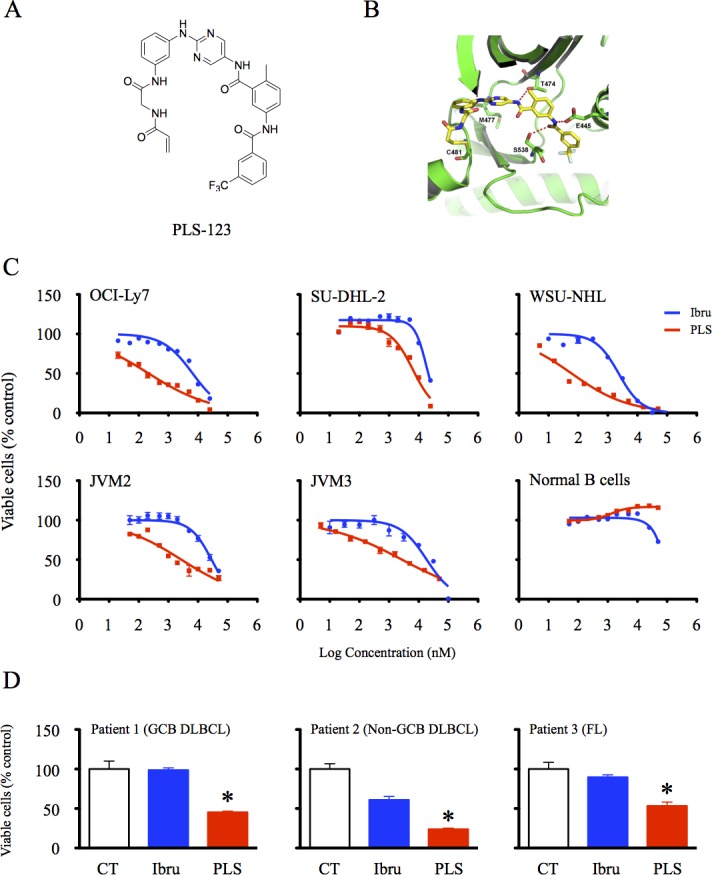
PLS-123 inhibited the viability of B-cell lymphoma cells effectively **A.** Chemical structure of novel Btk inhibitor PLS-123. **B.** Crystal structure of the human Btk in complex with PLS-123. **C.** B-cell lymphoma cell lines (OCI-Ly7, SU-DHL-2, WSU-NHL, JVM2 and JVM3) and CD19^+^ B cells from healthy volunteers (1 × 10^4^) were treated with the indicated concentrations of ibrutinib, PLS-123 or vehicle for 72 hours. **D.** Primary tumor cells (1 × 10^4^) from B-cell lymphoma patients were incubated with 1 μM ibrutinib, 1 μM PLS-123 or vehicle for 72 hours. The cell viability was determined using the Cell Titer-Glo luminescent cell viability assay. The results are expressed as the mean of relative activity and S.D. from triplicate cultures. *Significantly decreased compared with ibrutinib treatment (*p* < 0.05). The results are representative of at least three similar experiments.

PLS-123 is a novel high selective Btk inhibitor with an IC_50_ less than 5 nM (Figure 1A & 1B). The inhibitory activities of PLS-123 on the proliferation of 17 types of B-cell lymphoma cell lines were first analyzed using the cell viability assay. PLS-123 demonstrated enhanced anti-proliferative, dose-dependent effects compared with ibrutinib in 14 of these cell lines without affecting the viability of CD19^+^ B cells from healthy volunteers (Figure 1C). The GI_50_ values of Btk inhibitors, which were determined from dose-response curves, are presented in Table 1. The DLBCL cell line OCI-Ly7 and FL cell line WSU-NHL displayed the highest PLS-123 sensitivity with GI_50_ values in the double-digit nanomolar range. By contrast, the GI_50_ values for ibrutinib were in the micromolar range for these cells. Moreover, PLS-123 also more effectively suppressed the viability of primary tumor cells (Figure 1D). All these findings highlighted a remarkable anti-tumor activity of PLS-123 *in vitro*.

**Table 1:** Cytotoxicity effect of PLS-123 and ibrutinib towards B-cell lymphoma

Tumor type/cell line	GI_50_, Ibrutinib (μM)	GI_50_, PLS-123 (μM)
DLBCL		
OCI-Ly7	6.60	0.22
SU-DHL-2	18.38	6.35
SU-DHL-6	1.83	0.06
SU-DHL-16	6.31	0.81
Pfeiffer	0.014	0.016
OCI-Ly3	26.82	>50
FL		
WSU-NHL	2.32	0.06
RL	6.18	1.73
DOHH-2	0.52	0.02
CLL		
JVM3	18.67	2.74
MCL		
Mino	6.62	3.94
JVM2	28.68	2.75
JVM13	16.73	0.49
Jeko-1	20.43	>50
Granta519	21.12	2.57
Z138	19.24	15.88
Burkitt		
Namalwa	>50	26.48

### PLS-123-mediated B-cell lymphoma cytotoxicity is caspase-dependent

**Figure 2:**
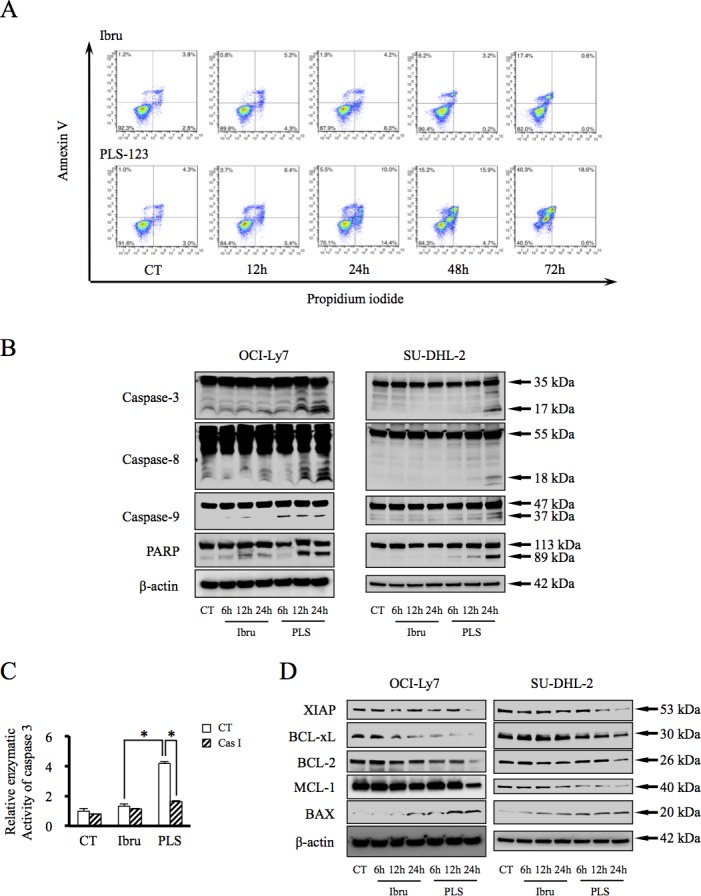
PLS-123-mediated B-cell lymphoma cytotoxicity is caspase-dependent **A.** OCI-Ly7 cells (1 × 10^6^) were cultured with 1 μM ibrutinib, 1 μM PLS-123 or vehicle for the indicated periods, and apoptotic cells were analyzed by flow cytometry. **B & D.** OCI-Ly7 and SU-DHL-2 cells (1 × 10^6^) were treated with 1 μM ibrutinib, 1 μM PLS-123 or vehicle for the indicated periods. Whole cell extracts were probed by Western blot for Caspase-3, Caspase-8, Caspase-9, PARP, XIAP, BCL-xL, BCL-2, MCL-1 and MAX. β-actin is shown as a loading control. **C.** OCI-Ly7 cells (1 × 10^6^) were pre-treated in presence or absence of the pan-caspase inhibitor z-VAD-fmk for 1 hour and then incubated with 1 μM ibrutinib, 1 μM PLS-123 or vehicle for 24 hours. Caspase-3 enzymatic activity was measured in tumor cells as described in the Materials and Methods. The results were expressed as the mean of the relative activity and S.D. from triplicate cultures. *Significantly changed compared with the control (*p* < 0.05). Results are representative of at least three similar experiments.

To investigate the mechanisms by which PLS-123 induced stronger cytotoxic effects than ibrutinib in malignant B cells, annexin V- and PI-stained apoptotic cells were analyzed by flow cytometry in the PLS-123-sensitive OCI-Ly7 cells. The percentage of annexin V-positive and PI-negative apoptotic cells peaked 24 hours after PLS-123 treatment (23.5%), whereas ibrutinib resulted in apoptosis in only 9.9% of the cells (Figure 2A). These findings correlated with the cell viability results shown in Figure 1. Furthermore, Western blotting analysis suggested that PLS-123 more strongly activated cleaved caspase-3, -8 and -9 as well as PARP compared with ibrutinib during apoptosis induction in OCI-Ly7 and SU-DHL-2 cells (Figure 2B). To confirm caspase-3 involvement in this apoptosis process, caspase-3 enzymatic activity was determined after treatment with PLS-123 and ibrutinib for indicated period. As shown in Figure 2C, the active form of caspase-3 was more significantly increased upon treatment with PLS-123 compared with ibrutinib. Meanwhile, PLS-123-induced caspase-3 cleavage was completely inhibited by the pan-caspase inhibitor z-VAD-fmk, thereby demonstrating that PLS-123 induced cytotoxicity against B-cell lymphoma cells is caspase-dependent.

The role of Bcl-2 family proteins in the regulation of caspase activation has been well characterized in the mitochondrial apoptotic pathway. The expressions of Bcl-2 family proteins were next analyzed by immunoblotting analysis. As shown in Figure 2D, PLS-123 treatment of malignant B cells resulted in dramatically decreased levels of anti-apoptotic proteins, such as XIAP, Bcl-2, Bcl-xL and Mcl-1. On the other hand, the pro-apoptotic protein BAX was significantly upregulated, thereby suggesting that PLS-123 could promote the apoptotic pathway via regulation of protein targets within mitochondria.

### PLS-123 precisely regulates the activation and catalytic properties of Btk, and results in greater attenuation of the BCR activating pathway than ibrutinib

**Figure 3:**
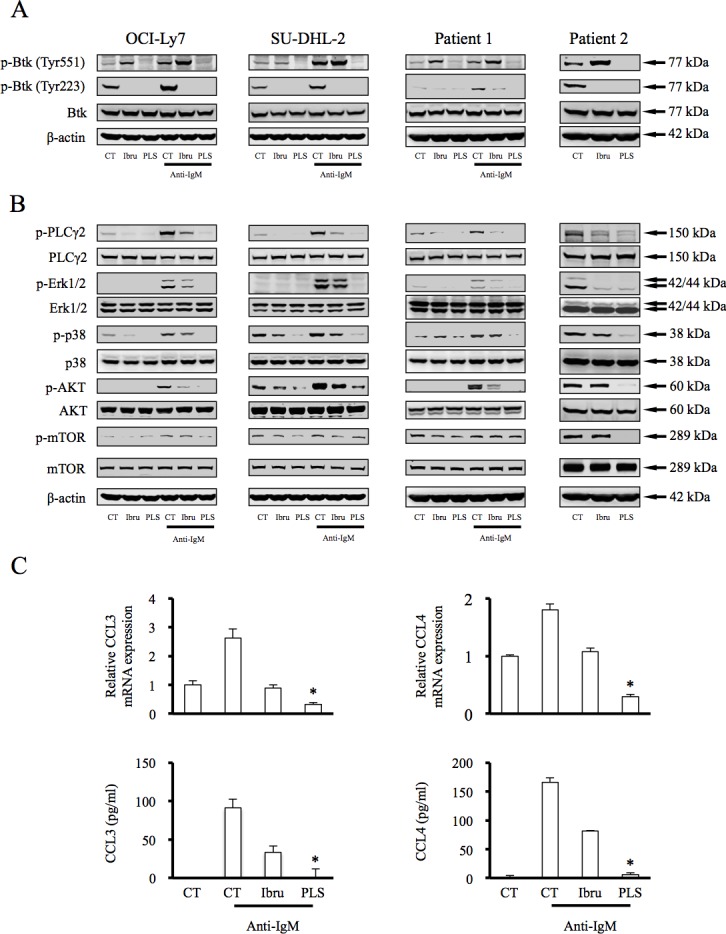
PLS-123 precisely regulates the activation and catalytic properties of Btk, and results in greater attenuation of the BCR activating pathway than ibrutinib **A, B.** OCI-Ly7, SU-DHL-2 and primary tumor cells (1 × 10^6^) were pretreated with 1 μM ibrutinib, 1 μM PLS-123 or vehicle for 1 hour and then stimulated or not with anti-IgM (5 μg/ml) for 10 minutes. Whole cell extracts were probed by Western blot for Btk, PLCγ2, ERK, p38, Akt and mTOR. β-actin is shown as a loading control. **C.** OCI-ly7 cells (1 × 10^6^) were pretreated with 1 μM ibrutinib, 1 μM PLS-123 or vehicle for 1 hour and then stimulated by anti-IgM (5 μg/ml) for 12 hours or not. CCL3 and CCL4 mRNA expression and cytokine production were detected by real time PCR and ELISA. *Significantly decreased compared with ibrutinib treatment (*p* < 0.05). The results are representative of at least three similar experiments.

As a novel Btk inhibitor, possible impacts of PLS-123 towards BCR signaling cascades were next investigated by immunoblotting analysis. The upstream BCR signal-activated kinases induce Btk phosphorylation at the Tyr551 residue, thereby resulting in a 10-fold increase in Btk's catalytic activity. This activity is followed by Btk auto-phosphorylation at Tyr223 and activation of downstream substrates. Ibrutinib inhibits the catalytic activities of Btk and the negative BCR pathway feedback loop, resulting in amplified phosphorylation at Tyr551 [11]. In our experiment, after 1 hour pretreatment with both Btk inhibitors, DLBCL cell lines and primary tumor cells were stimulated with anti-IgM to mimic BCR/antigen encounters and activate the BCR signal pathway. PLS-123 not only more significantly suppressed Btk phosphorylation at Tyr223 compared with ibrutinib but also reduced elevated Btk phosphorylation at Tyr551 (Figure 3A). Upon stimulation with anti-IgM, fully activated Btk coordinates PLCγ2 phosphorylation, thereby resulting in the activation of downstream cascades, including the MAPK and AKT/mTOR signaling pathways. Similar to inhibitory activity towards Btk phosphorylation, Western blotting analyses demonstrated that PLS-123 also effectively reduced PLCγ2, ERK1/2, p38, AKT and mTOR activation more than ibrutinib does (Figure 3B).

To further confirm the influence of PLS-123 towards BCR signaling pathway, CCL3 and CCL4 chemokine secretions from tumor cells after Btk inhibitor treatment, which is highly regulated and correlated with the signal activation of the BCR [15, 16], were measured via real-time PCR and ELISA. OCI-Ly7 cells were first pretreated with Btk inhibitors for 1 hour before activating the BCR signaling pathway to initiate CCL3 and CCL4 mRNA transcription. As shown in Figure 3C, both chemokines' mRNA and protein expressions were more dramatically reduced by PLS-123 treatment compared with ibrutinib, indicating that PLS-123 resulted in greater attenuation of the BCR activating pathway than ibrutinib. More importantly, all these results might also potentially account for the enhanced anti-proliferative activity of PLS-123 in B-cell lymphoma.

### Gene expression profile of B-cell lymphoma post PLS-123 treatment

**Figure 4:**
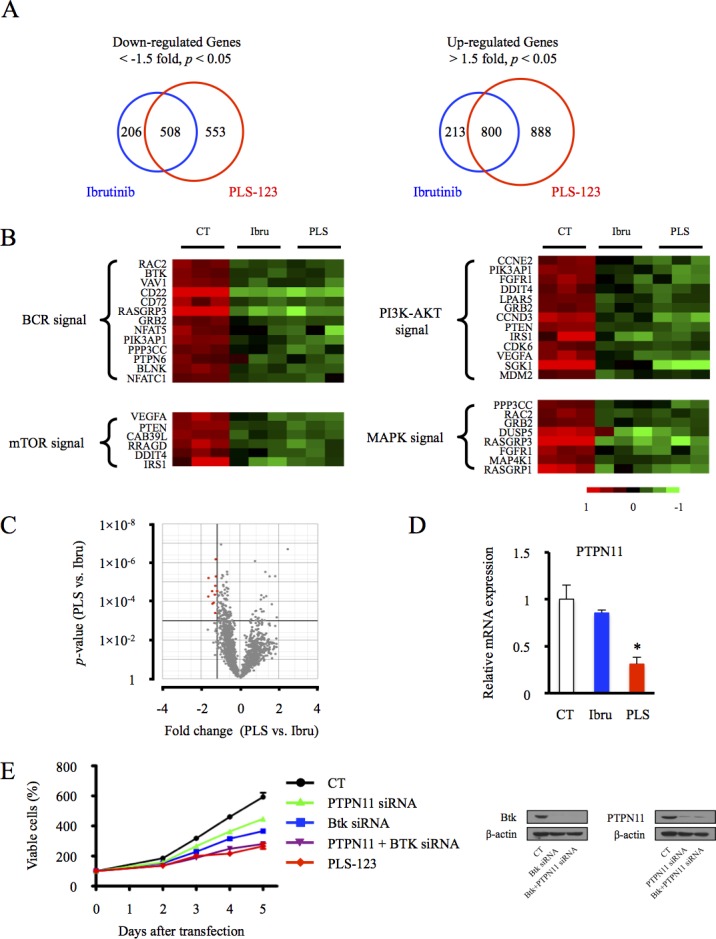
Gene expression profile of B-cell lymphoma post PLS-123 treatment **A.** OCI-Ly7 cells exposed for 24 hours with ibrutinib, PLS-123 or vehicle were submitted to gene expression profile analysis. Venn diagram depicting the top up and downregulated gene changes. **B.** Heatmap of the top representative downregulated genes associated with PLS-123 and ibrutinib treatment. **C.** The volcano plot depicts a log transformation plot of the fold difference (x-axis) and the *p* value (y-axis) of indicated genes between PLS-123 and ibrutinib treatment. **D.** The expressions of PTPN11 gene were further confirmed by real time PCR. **E.** The OCI-Ly7 cells were transfected with indicated siRNA oligos targeting Btk and PTPN11. The cell viability of tumor cells was determined using the Cell Titer-Glo luminescent cell viability assay. *Significantly decreased compared with ibrutinib treatment (*p* < 0.05). The results are representative of at least two similar experiments.

Due to the different selectivity profile of PLS-123 from ibrutinib [14], global gene expression profiling was needed to carry out to get a more comprehensive insight into alternative anti-tumor mechanisms of this novel Btk inhibitor. First, compared with vehicle treatment control, Venn diagram illustrated these Btk inhibitors top up- and down-regulated gene changes. Ibrutinib was less efficient with 714 transcripts significantly down (< −1.5 fold, *p* < 0.05) compared with 1061 for PLS-123. Interestingly, approximately 71% of transcripts (508 genes) downregulated by ibrutinib were also contained in the PLS-123 group (Figure 4A). Both inhibitors commonly top downregulated representative genes were illustrated by a heatmap in Figure 4B. Consistent with results from immunoblotting analysis in Figure 3, PLS-123 could efficiently downregulate BCR, AKT/mTOR and MAPK related signaling pathway.

On the other hand, the different selectivity profile of PLS-123 from ibrutinib might also contribute to distinct gene expression signatures and offer new opportunities beyond what ibrutinib has achieved. Volcano plots of 1441 genes changed in PLS-123 treatment alone (Down: 553 genes; Up: 888 genes) were then used to select additional anti-tumor target of the novel Btk inhibitor. When filter criteria were set for fold change < −1.5 and *p* < 0.001, 10 genes significantly decreased by PLS-123 were identified in Figure 4C. The mRNA expression analysis of these ten genes using real-time PCR further confirmed that PLS-123 led to significant downregulation of PTPN11 expression that might be due to the different selectivity profile of this inhibitor. As an important oncogenic gene in malignant B cells [17, 18], the inhibition of PTPN11 expression might also contribute to great efficacy of this novel Btk inhibitor. To demonstrate this hypothesis, PTPN11 and Btk knockdown effect on lymphoma cell viability as revealed by siRNA transfection. This co-transfection produced obvious anti-proliferative activity towards tumor cells, suggesting that PLS-123 might suppress tumor growth through combination Btk and PTPN11 inhibition (Figure 4E).

### PLS-123 overcomes BCR- and chemokine-mediated lymphoma cell adhesion and migration

**Figure 5:**
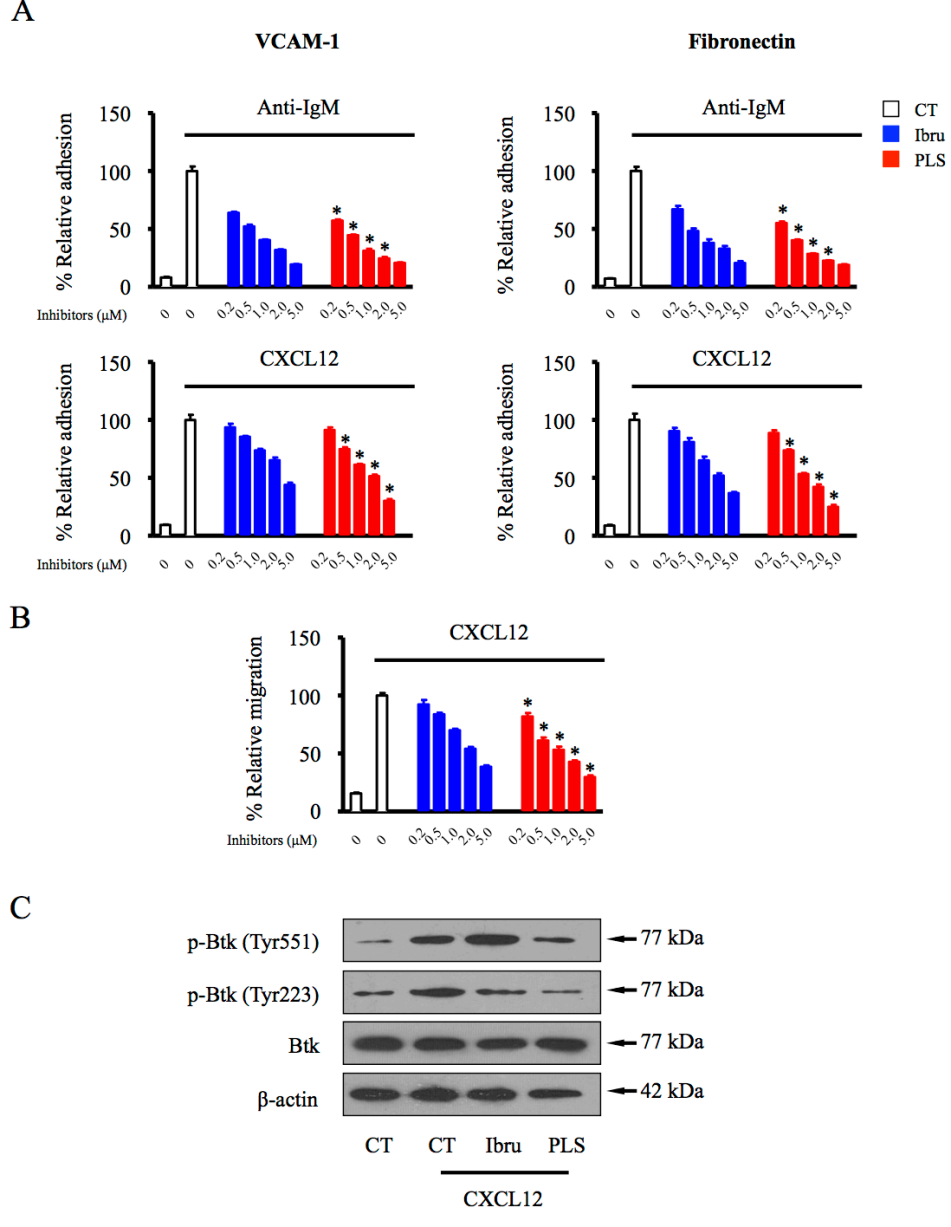
PLS-123 overcomes BCR- and chemokine-mediated lymphoma cell adhesion and migration **A.**Namalwa cells pretreated with increasing concentrations of ibrutinib, PLS-123 (0.2, 0.5, 1, 2 and 5 μM) or vehicle were stimulated with anti-IgM or CXCL12 for 30 mins, and then tumor cells were subjected to adhesion assays on plates precoated with fibronectin or VCAM-1. **B.** Namalwa cells were treated with increasing concentration of ibrutinib, PLS-123 (0.2, 0.5, 1, 2 and 5 μM) or vehicle and subjected to a chemotaxis migration assay in transwell plates with filters coated with VCAM-1, and CXCL12 was added into the lower chamber as a chemoattractant. **C.** Namalwa cells were pretreated with 1 μM ibrutinib, 1 μM PLS-123 or vehicle for 1 hour and then stimulated or not with CXCL12 for 10 minutes. Whole cell extracts were probed by indicated antibodies for Western blot analysis. *Significantly decreased compared with ibrutinib treatment (*p* < 0.05). The results are representative of at least three similar experiments.

B-NHL patients administered ibrutinib in clinical trials display reduced lymphadenopathy accompanied by substantial lymphocytosis, suggesting that the potential anti-adhesion activity of ibrutinib triggers B-lymphoid malignant cell mobilization from tumor tissues to the peripheral blood. To investigate possible effects of PLS-123 in active BCR- and chemokine-mediated adhesion, we analyzed anti-IgM- and chemokine CXCL12-induced adhesion to the extracellular matrix component fibronectin and the cellular adhesion molecule VCAM-1 in Namalwa cells. Compared with ibrutinib's inhibitory effects, our novel Btk inhibitor PLS-123 more significantly attenuated anti-IgM- and CXCL12-mediated adhesion to fibronectin and VCAM-1 in a dose-dependent manner (Figure 5A). Moreover, CXCL12 and its cognate receptor CXCR4 axis also appear to be crucial for migration and homing of malignant B cells via activation of BCR signal pathway [19]. In transwell culture system, PLS-123 also more efficiently blocked neoplastic cells migration toward chemokine CXCL12 through its dual-action inhibitory mode of Btk activation (Figure 5B & 5C).

### PLS-123 induces anti-tumor activities in B-cell lymphoma *in vivo*

**Figure 6:**
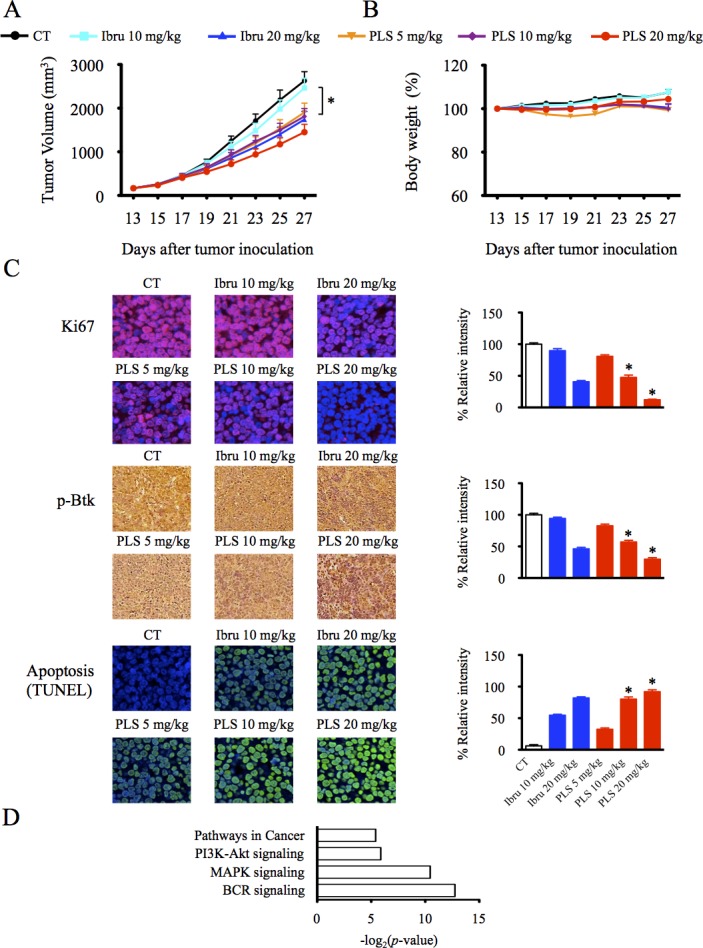
PLS-123 induces anti-tumor activities in B-cell lymphoma *in vivo* **A&B.** To generate tumors, CB.17/SCID mice were inoculated subcutaneously with 5 × 10^6^ OCI-Ly7 tumor cells in 0.1 ml PBS (each group contains 10 mice). PLS-123 and ibrutinib were administered to the tumor-bearing mice at the indicated concentrations when the tumor volume achieved a diameter of 150 mm^3^. Body weight and tumor volume of mice was measured every other day during treatment. **C.** Tumor tissue sections were stained as described under Materials and Methods with indicated antibodies for the determination of Btk inhibitors activity *in vivo*. *Significantly decreased or increased compared with ibrutinib treatment (*p* < 0.05). **D.** Total RNA extracted from tumor tissues of PLS-123 20 mg/kg and control group (n = 6 at each group) were applied to Affymetrix microarray. The top-ranking significant pathways downregulated by PLS-123 treatment were listed according to the *p* values (*p* < 0.05). The results are representative of at least two similar experiments.

The *in vivo* anti-tumor activity of the Btk inhibitors was examined in SCID mice inoculated with OCI-Ly7 cells. When the tumor volume was approximately 100-150 mm^3^, the mice were intraperitoneally administered the indicated concentrations of the Btk inhibitors. Fifteen days after administration, OCI-Ly7 tumor-bearing animals treated with 5 or 10 mg/kg of PLS-123 and 20 mg/kg ibrutinib resulted in significant effects blocking tumor growth. Similar to previous results from *in vitro* models, PLS- 123 also demonstrated more anti-growth effects than ibrutinib at the same drug dosage of 10 mg/kg (*p* = 0.038). Mice treated with 20 mg/kg of PLS-123 displayed the strongest suppressive activities, and this dosage induced 45% tumor reduction without significant effects on body weight (*p* = 0.418; Figure 6A & 6B).

To further evaluating the proliferation, apoptosis and BCR signal status of tumor tissue post administration of Btk inhibitors, Ki-67, TUNEL and p-Btk immunohistochemistry staining was performed on paraffin sections. As shown in Figure 6C, PLS-123 treatment produced a more significant decrease of Ki-67 and p-Btk expression than the same concentration of ibrutinib achieved. Moreover, activation of apoptosis was also clearly observed post PLS-123 treatment, as indicated by an increase in TUNEL staining. Meanwhile, total RNA extracted from tumor tissues of PLS-123 20 mg/kg and control group were applied to gene expression microarray. Pathway analysis of microarray data confirmed that PLS-123 mostly down-regulated BCR, MAPK and PI3K-Akt signaling pathway *in vivo* (Figure 6D). Collectively, all these results confirmed our *in vitro* findings, in which PLS-123 induced lymphoma cell death via inhibition of BCR signaling and induction of apoptosis.

## DISCUSSION

In this paper, we focused on the anti-tumor activities of the novel Btk inhibitor PLS-123 in B-cell lymphoma. PLS-123′s potent anti-proliferative and pro-apoptotic effects in a wide range of B-cell lymphoma subtypes may be attributed to dual-action Btk inhibitory mode and alternative selectivity profile provided additional anti-tumor mechanisms.

Reports implicated that various B-cell subsets utilize qualitatively distinct BCR signaling to promote their own growth and survival. Lam et al first reported that ablation of BCR expression resulting in a rapid death of peripheral B cells, indicating the requirement for this tonic BCR signaling to sustain the viability of B cells [20]. Tonic BCR signaling was also suggested to sustain the survival of Burkitt lymphoma through engagement of the PI3K pathway [21]. Ibrutinib and our compound PLS-123 are very potent Btk inhibitors, yet exhibit no inhibitory role towards Burkitt lymphoma and normal B cells, suggesting these compounds might not be efficient targeted approaches towards tonic BCR signaling and Btk is not essential for tonic BCR signaling. Some lymphoma subtypes, such as ABC-DLBCL, FL, CLL and MCL, maybe are more akin to rely upon chronic BCR signaling [3, 22]. Ibrutinib has shown great efficacy in treating MCL and CLL patients yet its anti-proliferative activity is inferior to PLS-123. If these cell lines do rely more on the chronic BCR signaling pathway, PLS-123 could have a better therapeutic effect in the clinic. As already suggested in the results section, the constitutively activated Btk and PLCγ2 could be detected not only in non-GCB but also in several GCB-DLBCL (Figure 3A, 3B and data not shown). It is in accordance to literature reports that some GCB-DLBCL relied on activated NF-κB, which indicated that chronic active BCR signaling mechanisms might involve in the pathogenesis of these patients [23, 24]. Thus, it is not surprising that the novel Btk inhibitor PLS-123 exhibits anti-tumor activity in some GCB-DLBCL.

Btk is a member of Tec family non-receptor protein-tyrosine kinases, which participates in biological events through its catalytic ability and protein-protein interactions. When ibrutinib was used, activated Btk maintained at the Tyr551 phosphorylated stage and did not come back to the resting form, thus it could still engage into the signaling complexes or other functions through protein-protein interactions. Kurosaki et al also reported that phosphorylation of Tyr551, but not Tyr223, is the key regulatory position for BCR signaling activation using Btk-deficient DT40 cell line expressing Btk (Y223F) and Btk (Y551F) mutation models [25]. Thus, in contrast to ibrutinib, which only inhibits Btk's catalytic activity at Tyr223, our novel developed Btk inhibitor PLS-123 abolished Btk's activation via transphosphorylation at Tyr551 and subsequent autophosphorylation at Tyr223, which resulted in a more concise and effective regulation of BCR signal and significant anti-proliferative activities in malignant cells. This dual-action inhibitory mode of PLS-123 could be considered as a simultaneous application of a Btk inhibitor and its upstream kinase inhibitor to doubly control the BCR signal pathway. One advantage of this approach is that it does not need to care about which kinase (Lyn, Syk or both) is the primary Btk activator(s).

Enzymatic activity screen showed that ibrutinib is not a very selective inhibitor, but promising clinical efficacy was observed in relapsed/refractory B-cell lymphoma patients, indicating that pharmacological effects through other pathways in addition to inhibition of Btk may function here. Thus, it was very valuable to present a new series of Btk inhibitors that has a different mode of regulation and a different selectivity profile to this field. The alternative selectivity profile of our novel Btk inhibitor might offer new anti-tumor mechanisms and potent inhibitory activities in a wider range of B-cell lymphoma subtypes. In our experiment, the microarray results demonstrated approximately 71% of transcripts downregulated post ibrutinib treatment was also similarly attenuated by PLS-123. Moreover, the oncogenic gene PTPN11, which was significantly decreased by PLS-123, was identified as an additional anti-tumor mechanism of this novel Btk inhibitor. It is reported that the hyperactivation and misregulation of PTPN11 played an oncogenic role in various tumor types, especially germinal center derived lymphoma [18, 26, 27]. In our study, the combination Btk and PTPN11 inhibition may synergistically suppress growth of malignant B cells (Figure 4E). This notion indicated that PLS-123′s great efficacy and anti-proliferative effects in GCB-DLBCL lymphoma might not only be simply attributed to downregulation of BCR signaling pathway alone and alternative selectivity profile provided downregulation of PTPN11 expression should also be considered. However, further experimentation is still needed to explore the precise mechanism.

Caspases are a family of proteases that are frequently activated in mammalian cell apoptosis. Ibrutinib and R406 inhibited proliferation and induced apoptosis in CLL and DLBCL via caspase activation [28-30]. In our study, we found that PLS-123 efficiently induced the activation of caspases and regulated Bcl-2 family members expression, suggesting that PLS-123 potentially activated the mitochondrial apoptotic pathway in a caspase-dependent manner. Moreover, it is well demonstrated that the BCR signaling pathway can sustain the expression of anti-apoptotic proteins through activation of the PI3K/Akt and MEK/ERK pathways [31], supporting the notion that PLS-123 suppressed the expression of these proteins via inhibition of downstream BCR-activating pathways.

The interaction of malignant B cells and surrounding tumor microenvironment is thought to contribute to the survival and progress of B-cell malignancies [32, 33]. The encouraging clinical efficacy of ibrutinib not only results from direct cytotoxic effects but also its inhibition of the cellular adhesion and migration between tumor cells and the surrounding microenvironment [34-37]. After ibrutinib treatment, most CLL and MCL patients display a transient increase in circulating lymphocytes and a reduction in spleen and lymph node size, indicating the anti-adhesion activity of ibrutinib shifts the malignant cells from the infiltrated tissue to the peripheral blood. In our study, anti-IgM and chemokine treatment increased the adhesion of B-NHL cells to cellular substrates, such as fibronectin and VCAM-1; this activity was dramatically inhibited by PLS-123 in a dose-dependent manner (Figure 5A), suggesting the essential impact of novel Btk inhibitor towards crosstalk between malignant cells and the microenvironment.

Collectively, our novel dual-action Btk inhibitor PLS-123 suggested a new direction to pharmacologically modulate Btk function and develop potentially therapeutic drug for the treatment of B-cell lymphoma.

## MATERIALS AND METHODS

### Cell lines, patient samples and culture conditions

The human DLBCL cell lines (OCI-Ly7, SU-DHL-16 and OCI-Ly3) and MCL cell lines (JVM2, JVM13, Granta519, Mino, Z138 and Jeko-1) were kindly provided by Dr. Fu (University of Nebraska Medical Center, Omaha, NE, USA). SU-DHL-2, SU-DHL-6, Pfeiffer, RL, DoHH2, WSU-NHL, JVM-3, and Namalwa cells were obtained from ATCC (Manassas, VA) and DSMZ (Braunschweig, Germany). All cells were grown in IMDM or DMEM supplemented with 10% fetal bovine serum (FBS; Gibco, Life Technology), L-glutamine and penicillin-streptomycin in a humidified atmosphere of 5% CO_2_ at 37°C. The authentication of all cell lines was performed using short tandem repeat DNA fingerprinting analysis (Applied Biosystems, Foster City, CA).

B-cell lymphoma biopsy specimens were collected under a protocol that was approved by the Institutional Review Board of the Peking University Cancer Hospital & Institute, and informed consent was obtained from all patients. Fresh biopsy specimens were cut into small pieces and gently minced over a wire mesh screen to create a cell suspension. Mononuclear cells were isolated via density gradient centrifugation over Lymphoprep (Axis Shield, Oslo, Norway). CD19^+^ primary tumor cells were purified using negative selection with B-cell Isolation Kit (Miltenyi Biotec, Bergisch-Gladbach, Germany). CD19^+^ cell purity was typically greater than 95% as determined by FACS analysis (BD FACS Aria II, BD Bioscience, NJ, USA).

### Reagents and antibodies

The Btk inhibitors ibrutinib and PLS-123 were synthesized at the laboratory of Dr. Zhengying Pan at Peking University Shenzhen Graduate School according to a previously published procedure [14, 38]. Antibodies against Caspase-3 (#9662), Caspase-8 (#9746), Caspase-9 (#9508), XIAP (#2045), BCL-xL (#2764), BCL-2 (#2870), MCL-1 (#5453), BAX (#5023), phospho-Tyr223-Btk (#5082), Btk (#8547), phospho-Tyr759-PLCγ2 (#3874), phospho-Tyr1217-PLCγ2 (#3871), PLCγ2 (#3872), phospho-p38 (#9211), p38 (#8690), phospho-Thr308-AKT (#9275), AKT (#9272), phospho-Ser2448-mTOR (#2971), mTOR (#2972), phospho-ERK1/2 (#4370) and ERK1/2(#9102) were obtained from Cell Signaling Technology (Danvers, MA, USA). The anti-PARP, phospho-Tyr551-Btk, β-actin (A5441), IgM, Ki-67 antibodies were obtained from BD Biosciences, Sigma (St. Louis, MO, USA) and Abcam (Cambridge, MA).

### Cell viability assay and apoptosis detection

Cell viability and apoptosis was measured using the Cell Titer-Glo luminescent cell viability assay system (Promega, Madison, WI, USA), Annexin V/PI apoptosis detection kit (BD Biosciences), Active Caspase-3 Quantikine ELISA kit (R&D Systems Inc., Minneapolis, MN, USA) and in situ cell death detection kit (Roche, Germany) according to the manufacturer's instructions.

### Western blotting

To prepare whole cell extracts, OCI-Ly7 and SU-DHL-2 cells were lysed with RIPA lysis buffer [39]. Equivalent amounts of protein (10 μg) were resolved on SDS-PAGE gels, transferred and immobilized on nitrocellulose membranes (Amersham, Buckinghamshire, UK) and probed with the appropriate primary and secondary antibodies. Immunodetection was performed using a chemiluminescence detection system (Alpha Innotech, San Leandro, CA, USA).

### Real-time quantitative PCR

OCI-Ly7 cells were treated with Btk inhibitors in the presence or absence of anti-IgM for 12 hours, and total RNA was extracted via RNA TRIzol Extraction (Life Technologies). The primer and probe sequences of the target genes are listed as follow: β-actin, 5′-CCTGGCACCCAGCACAAT-3′, 5′-GCCGATCCACACGGAGTACT-3′ and 5′-ATCAAGATCATTGCTCCTCCTGAGCGC-3′; CCL3, 5′-GAGCCCACATTCCGTCACCT-3′, 5′-CACTGGCTGCTCGTCTCAAA-3′ and 5′-CCACTGCTGCCCTTGCTGTCC-3′; CCL4, 5′-CAGCGCTCTCAGCACCAA-3′, 5′-AGCTTCCTCGCAGTGTAAGAAAA-3′ and 5′-CTCAGACCCTCCCACCGCCTGC-3′.

### ELISA

CCL3 and CCL4 production from tumor cells was detected using Human CCL3 and CCL4 Quantikine ELISA Kits (R&D Systems).

### Adhesion and migration assay

Namalwa cells pretreated with the indicated concentrations of Btk inhibitors or vehicle were stimulated with anti-IgM or CXCL12 for 30 mins, and then cells were seeded into 96-well plates that were coated with fibronectin (BD Biosciences) or VCAM-1 (Sigma). Thirty minutes later tumor cells were washed twice with PBS and adherent cells were measured using the Cell Titer-Glo luminescent cell viability assay system. Migration assays were performed in triplicate with transwell insert chambers coated with VCAM-1. Namalwa cells treated with Btk inhibitors or not in the upper chamber were allowed to migrate towards lower compartment contained CXCL12 (R&D Systems) for 4 hours. The migration of control untreated cells in the presence of CXCL12 was normalized to 100%.

### Microarray hybridization and gene expression analysis

Sample processing, microarray hybridization and gene expression analyses were performed using the Affymetrix GeneChip System (Affymetrix, Santa Clara, CA, USA). The biotinylated cRNA was fragmented and hybridized to the GeneChip Human Genome U133 Plus 2.0 microarrays (Affymetrix). Pathway analysis was used to identify the significant pathway of the differential genes according to KEGG database [40-42]. The Raw data have been deposited in the Gene Expression Omnibus Database (GSE65816 and GSE65817).

### siRNA transfection

OCI-Ly7 cells were seeded on 24-well plates in complete media on the day of the transfection. siRNA oligonucleotides targeting PTPN11 (5′-GAAGCACAGUACCGAUUUATT-3′), Btk (5′-GGCAGUAAGAAGGGUUCAATT-3′) were synthesized by Invitrogen and transfected to cells using the Lipofectamine^TM^ RNAiMAX reagent (Life Technologies). The efficiency of siRNA knockdown of target genes was determined by Western blotting.

### *In vivo* studies

Six- to eight-week-old male CB.17/severe combined immunodeficiency disease (SCID) mice were inoculated subcutaneously with 5 × 10^6^ OCI-Ly7 tumor cells in 0.1 ml PBS for tumor development. Treatments were initiated when the tumor volume achieved approximately 100-150 mm^3^. According to the dose of ibrutinib widely used *in vivo* experiment [35], the mice were administered vehicle or 5, 10 or 20 mg/kg of Btk inhibitors intraperitoneally qd for 15 days. The tumor tissue samples were collected from all the 6 groups at 4 hours after the last dosing. All animal experiments were performed according to the guidelines for the care and use of animals, which were approved by the Peking University Cancer Hospital & Institute.

### Immunohistochemistry and Immunofluorescence analysis

Immunohistochemistry stains for p-Btk were performed in the department of pathology of Peking University Cancer Hospital using the standard streptavidin-biotin-peroxidase immunostaining procedure. For Ki-67 immunofluorescence analysis, paraffin sections were dewaxed, rinsed, and blocked with 2% BSA for 20 mins. Then slides were incubated with anti-Ki-67 antibody for 2 hours and followed with fluorescein-conjugated secondary antibodies for another 2 hours. DAPI was used for nuclear counterstaining. Slides were mounted with 90% glycerol in PBS and visualized with a laser scanning confocal microscopy (TCS-SP2, Leica Microsystems).

### Statistical analysis

All experiments were repeated more than three times and representative results are shown in the figures. Results are expressed as mean ± SD. Statistical analysis was performed using Student's *t* test. A confidence level of *p* < 0.05 was considered significant.
